# Targeted Sequencing of Germline Breast Cancer Susceptibility Genes for Discovering Pathogenic/Likely Pathogenic Variants in the Jakarta Population

**DOI:** 10.3390/diagnostics12092241

**Published:** 2022-09-16

**Authors:** Sonar Soni Panigoro, Rafika Indah Paramita, Kristina Maria Siswiandari, Fadilah Fadilah

**Affiliations:** 1 Surgical Oncology Division, Department of Surgery, Faculty of Medicine, Universitas Indonesia, Central Jakarta 10430, DKI Jakarta, Indonesia; 2 Doctoral Program in Biomedical Sciences, Faculty of Medicine, Universitas Indonesia, Central Jakarta 10430, DKI Jakarta, Indonesia; 3 Department of Medical Chemistry, Faculty of Medicine, Universitas Indonesia, Central Jakarta 10430, DKI Jakarta, Indonesia; 4 Bioinformatics Core Facilities—IMERI, Faculty of Medicine, Universitas Indonesia, Central Jakarta 10430, DKI Jakarta, Indonesia

**Keywords:** breast cancer, germline variants, NGS, sequencing, young women

## Abstract

Germline predisposition plays an important role in breast cancer. Different ethnic populations need respective studies on cancer risks pertinent to germline variants. We aimed to discover the pathogenic and likely pathogenic variants (P/LP-Vs) of germline breast cancer susceptibility genes and to evaluate their correlation with the clinical characteristics in Jakarta populations. The pure DNA was extracted from the blood buffy coat, using reagents from the QIAamp DNA Mini Kit^®^ (Qiagen, Hilden, Germany). The DNA libraries were prepared using the TargetRich™ Hereditary Cancer Panel (Kailos Genetics^®^, Huntsville, AL, USA). The barcoded DNA libraries were sequenced using the Illumina NextSeq 500 platform. In-house bioinformatics pipelines were used to analyze the gene variants. We identified 35 pathogenic and likely pathogenic (P/LP-Vs) variants (28 frameshift, 5 nonsense, and 2 splice-site variants). The P/LP-Vs group was statistically significantly different in luminal B status (*p* < 0.05) compared with the non-P/LP-Vs group. The P/LP-Vs found both in BRCA1/2 genes and non-BRCA genes may increase the risk of breast cancer and alter drug responses. The screening of multigene variants is suggested, rather than BRCA testing only. Prior knowledge of the germline variants status is important for optimal breast cancer diagnosis and optimal therapy.

## 1. Introduction

Among developing nations, breast cancer is the leading cause of mortality and it is the most prevalent type of cancer in women globally. Globally, 2.3 million cases were recorded by GLOBOCAN in 2020, representing the fifth cause of cancer-related mortality, with cases in Asia being higher than for any other continent. By 2020, breast cancer continued to be the most common new case of cancer in women (30.8%) and was the leading cause of death in Indonesia (15.3%) [1].

A new method of genetic study has emerged with the development of next-generation sequencing (NGS) technology. For screening individual genes and small gene panels in clinical settings, NGS has established itself as an alternative, cost-effective tool. The finding of the susceptibility genes linked to cancer risk was accelerated by NGS in the late 2000s [2]. Compared with BRCA1/2 testing alone, multi-gene panel testing could significantly uncover the cancer risk gene variations in more patients [3].

Breast cancer is the most prevalent type of cancer in women, and inherited predisposition is a significant factor. Better information and comprehension of the underlying genetic factors would thus be beneficial for the management and prevention of breast cancer [4]. Practice recommendations for breast cancer will be influenced by knowledge of the prognosis following a diagnosis and the risk assessment of genetic mutations of cancer susceptibility. Many clinical trials are currently evaluating the use of poly (ADP-ribose) polymerase (PARP) inhibitors in the treatment of breast cancer because of their effectiveness in controlling BRCA mutation positive tumors. It is anticipated that these agents will be incorporated into systemic therapy in clinical practice. As a result, systemic therapy for BRCA mutation-associated breast cancer has been made based on the presence of BRCA mutations rather than the disease features only [5].

The genes BRCA1, BRCA2, PALB2, TP53, ATM, CHEK2, STK11, and NBN have all been identified as breast cancer risk genes [6]. BRCA1, BRCA2, CDH1, PTEN, STK11, and TP53 are examples of high-penetrance genes, while ATM, CHEK2, BARD1, and RAD51D are examples of moderate-penetrance genes [7]. Other research has shown that harmful variations in RAD51C, which is crucial for HR repair, increase the chance of developing breast and ovarian cancer [8]. The DNA mismatch repair genes (MLH1, MSH2, MSH6, and PMS2), which are germline variants that are mostly linked to Lynch syndrome, have been found to be correlated with breast cancer risk or survival [5]. It is interesting to note that Chinese and Japanese populations have pathogenic BRIP1 variants that have not been found in Western populations. The carrier frequency of the detrimental PALB2 mutation is different from that in Western populations, similar to the BRIP1 mutation. This variation might be caused by the ethnicity of the genome [8]. We investigated the germline variant linked to breast cancer risk and outcomes in our cohort using gene panels in order to better understand these variants.

It is necessary to conduct individual research in various ethnic populations in order to determine cancer risks related to germline variations. The autosomal dominant conditions most frequently linked to a high risk of breast cancer are hereditary breast and ovarian malignancies brought on by mutations in BRCA1 or BRCA2. Numerous research in Asian groups revealed findings that were distinct from those found in Caucasian people [5,9,10]. In contrast with Western countries, where the incidence of breast cancer rises among postmenopausal women in their 60s, the incidence of breast cancer peaks in premenopausal women in their 40s in Asian countries [11]. Asian patients may have breast cancer at a younger age than their Caucasian peers because their BRCA1/2 mutations are thought to have different contributions than those of Caucasians [12]. Enhancing the database of variants in various ethnic communities is essential for improving the interpretation of ethnically specific germline variants and the management of cancer risks in ethnically appropriate populations. In this work, we sought to identify pathogenic and likely pathogenic variants (P/LP-Vs) of germline genes associated with breast cancer susceptibility and to assess their association with clinical traits in the Jakarta population.

## 2. Materials and Methods

### 2.1. Patients

The study was approved by the Ethical Committee at Faculty of Medicine Universitas Indonesia on 16 October 2017 (approval number: 958/UN2.F1/ETIK/2017). All procedures were carried out in conformity with the applicable rules and regulations. Before the patients’ samples were taken, all of the patients gave their informed consent for this study. Through participant surveys and computerized medical records, clinical data were gathered.

A total of 75 female breast cancer patients under the age of 45 who were receiving treatment at the Surgical Oncology Division, Department of Surgery, Cipto Mangunkusumo National Hospital Jakarta were included in this study. For the purposes of this analysis, all patients who had breast cancer as determined by histology and immunohistochemistry between January 2014 and December 2017 were eligible. Patients with incomplete histology and medical record information were disqualified from this investigation. None of the participants knew their gene variations associated with cancer risk prior to being recruited. Distant metastases and stage grouping classification were defined according to the American Joint Committee for Cancer (AJCC) 7th Edition [13]. Molecular subtypes (Luminal A, Luminal B, HER2, and triple-negative) were defined based on hormone receptors and HER2 status.

### 2.2. DNA Extraction, Library Preparation, and Sequencing

Pure DNA was extracted from the blood buffy coat, using reagents from the QIAamp DNA Mini Kit^®^ (Qiagen, Hilden, Germany). TargetRichTM Hereditary Cancer Panel (Kailos Genetics^®^, Huntsville, AL, USA) was used to create the DNA libraries. This panel is 22 genes targeted-sequencing panel (Table S1). TargetRichTM UMI/Index Adapter Plate (Kailos Genetics^®^, Huntsville, AL, USA) was combined with DNA libraries to enable sample barcoding, multiplex sequencing, and tagging of individual collected DNA molecules. The Illumina NextSeq 500 platform was used for the sequencing of the barcoded DNA libraries. All of the isolation and genome sequencing methods of 75 samples were described in Panigoro et al., 2020 [14].

### 2.3. Bioinformatics Analysis

Using FastQC 0.11.9 [15], the paired-end raw readings from each sample were examined for quality. After quality control, using the Trimmomatic [16] program, all of the low-quality reads were eliminated before the alignment phase. The BWA algorithm was used to align the fastq files with the hg38 human reference genome (Genome Reference Consortium GRCh38), which was retrieved from ENSEMBL (http://asia.ensembl.org/Homo_sapiens/Info/Index (accessed on 5 January 2022)) [17]. With the use of the Genome Analysis Toolkit (GATK, version 4.1.9) [18], the base quality score was recalculated. HaplotypeCaller from GATK (4.1.9 version) was used for the variant calling methods. The variant calling file output then went to the hard-filtering process using specific thresholds and throwing out any variants that had annotation values above or below the set threshold. The thresholds for SNV were QualByDepth (QD) > 2.0, FisherStrand(FS) < 60, StrandOddsRatio (SOR) < 3, and RMSMappingQuality (MQ) > 40.0. The thresholds for MNV were QualByDepth (QD) > 2.0, FisherStrand(FS) < 200.0 and StrandOddsRatio (SOR) < 10.0.

All of the variants were annotated using snpEff [19] (GRCh38.101 annotation database version), and snpSift (dbSNP database (http://www.ncbi.nlm.nih.gov/SNP/ (accessed on 10 January 2022)) annotated the variants with synonymous, missense, nonsense, frameshift, and splicing variants; ClinVar databases (http://www.ncbi.nlm.nih.gov/clinvar/ (accessed on 10 January 2022)) classified the variants as follows: 0—uncertain significance, 1—not provided, 2—benign, 3—likely benign, 4—likely pathogenic, 5—pathogenic, 6—drug response, 7—histocompatibility, and 255—other. The impact of novel variants on the protein function or structure was analyzed using VarSome [20], an integrated search engine that allows to access multiple databases, prediction tools, and publications at a single site. Nonsense, frameshift, and splice-site variants that resulted in a truncated protein product were classified as pathogenic.

SnpSift from the SnpEff package was used to filter the raw variants. Filtered variants were exported from variant files into tab-delimited files using SnpSift and were concatenated into a single tab-delimited file including all variants of all patients. Variants were visualized using Lollipop plot by MutationMapper (https://www.cbioportal.org/mutation_mapper (accessed on 2 February 2022)) [21,22]. The raw data of all samples were deposited in the SRA under BioProject number: PRJNA606794 (https://www.ncbi.nlm.nih.gov/bioproject/?term=PRJNA606794 (accessed on 25 December 2021)). 

### 2.4. Clinical Correlation and Statistical Analysis

For the study participants who previously had breast cancer, age of onset and familial history were taken from participant survey. Using the Chi-square test or t test, statistical correlations between the clinical features and P/LP-Vs or non-P/LP-Vs status were assessed. *p*-values and 95% confidence intervals were used to show statistical significance. To reject the null hypothesis, an alpha level of 0.05 was deemed statistically significant different. Prism 9.0.0 software was used for all of the analyses (trial version).

## 3. Results

### 3.1. Patients’ Characteristics

There were 75 patients aged ≤45 years at diagnosis, among whom 18 patients were included with familial breast cancer (mother or aunt or grandmother had breast cancer), and the remaining 57 patients were women without a familial history of breast cancer. The clinical characteristics of the patients are shown in Table 1. The mean age of the breast cancer group was 34 years. Regarding tumor histopathology, patients with familial breast cancer exhibited stage I (4; 22.2%), II (5; 27.8%), III (3; 16.7%), and IV (6; 33.3%), whether in sporadic patients exhibited stage I (9; 15.8%), II (22; 38.6%), III (16; 28.113%), and IV (10; 17.5%). 

### 3.2. Characteristics of the Germline Variants

**Figure 1:**
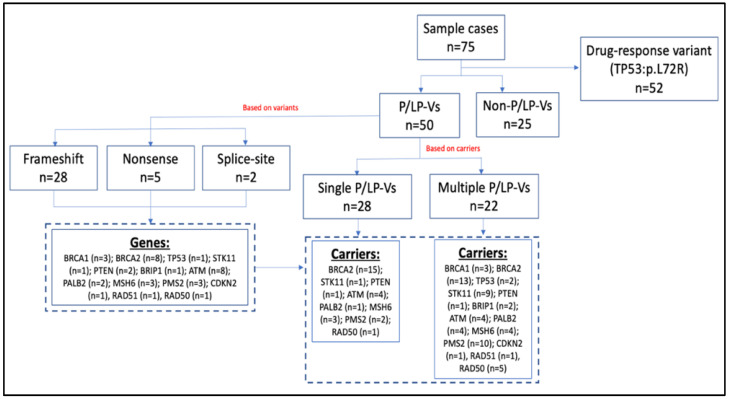
Flow chart of the germline variants characteristics.

In this study, we sequenced the established breast cancer susceptibility genes in 75 breast cancer patients. We annotated the variants based on the dbSNP and ClinVar databases. Novel variants that had not yet been found in the public databases were predicted using VarSome software. Pathogenic mutations included nonsense, frameshift, and splice-site variations that lead to shortened protein products. A flow chart of germline variant characteristics is shown in Figure 1. 

From Figure 1, we could identify 50 patients (67%) that carried P/LP-Vs. Based on the carriers, there were 28 (56%) of P/LP-Vs patients carried single P/LP-Vs and the remaining 22 patients (44%) carried multiple P/LP-Vs (Table S2). Based on the gene variants, we found 35 P/LP-Vs (Table 2) that contained 28 frameshift, 5 nonsense, and 2 splice-site variants. 

**Figure 2:**
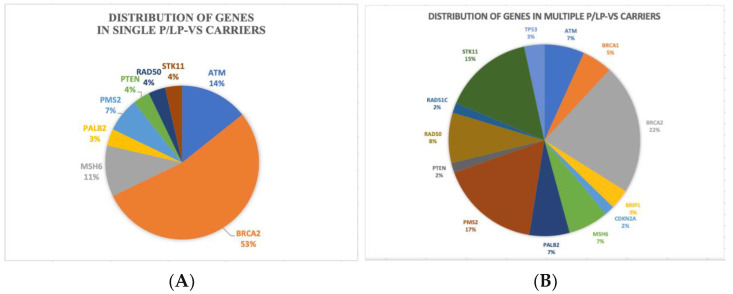
Distribution of genes in: (**A**) single P/LP-Vs carriers, (**B**) multiple P/LP-Vs carriers.

In the distribution of genes in single and multiple P/LP-Vs diagram (Figure 2), BRCA2 was the most predominant in both of the groups, with 53% and 22% in single P/LP-Vs carriers and multiple P/LP-Vs carriers (Table S2), respectively. Potentially pathogenic VUS missense variants were also found in this study. We identified 31 variants of uncertain significance (VUS), of which most of the variants had been recorded in the dbSNP database (Table S3).

**Figure 3:**
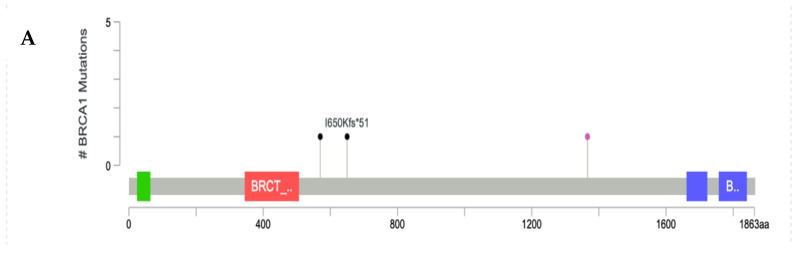

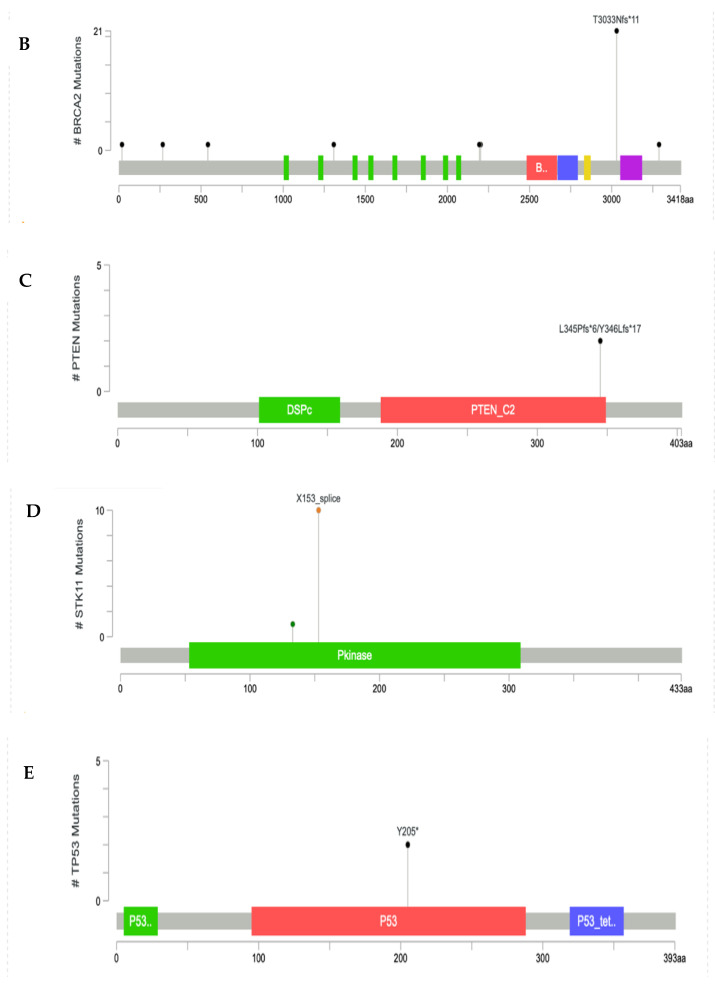
Lollipop plot of P/LP-Vs in high-penetrance genes: (**A**) BRCA1 gene, (**B**) BRCA2 gene, (**C**) PTEN gene, (**D**) STK11 gene, and (**E**) TP53 gene. *: terminated protein.

The positions of the pathogenic variants for high-penetrance genes, such as BRCA1, BRCA2, PTEN, STK11, and TP53, were visualized in Lollipop plot, as shown in Figure 3. Most of the P/LP-Vs had not been recorded yet in the dbSNP or CinVar databases, and were designated as novel variants with their clinical significance predicted by VarSome.

### 3.3. Prediction of Drug Response Alteration Associated with Gene Variants

There were some variants that showed alterations to drug therapy, based on the Onco-KB database prediction (Table 2). Patients with certain P/LP-Vs were predicted to benefit from targeted therapy. P/LP-Vs in BRCA1/2, BRIP1, ATM, PALB2, and ATM genes (23 of 36 P/LP-Vs) were predicted to benefit from the PARP inhibitor (Talazoparib or Olaparib). We also found one frameshift variant in CDKN2A that could be sensitive to CDK4/6 inhibitors such as Palbociclib, Ribociclib, and Abemaciclib [23,24]. Related to two variants in the PTEN gene, we found that loss-of-function variants could be sensitive to beta-isoform-selective PI3K-targeted inhibitors, such as the investigational agents GSK2636771 and AZD8186 [25]. Besides P/LP-Vs in several genes, there was a missense variant of TP53:p.P72R (dbSNP ID: rs1042522) in 52 patients (69.3%) that resistance to neoadjuvant chemotherapy, such as 5-fluorouracil and cisplatin.

### 3.4. Correlation of Variants with Tumor Characteristics

To characterize the clinical features upon cancer diagnosis in the germline variant carriers, we compared clinical characteristics between the pathogenic and likely pathogenic variant (P/LP-Vs) group and non-P/LP-Vs group (Table 3). None of the patients had prior knowledge of cancer susceptibility gene variant status at the time of their breast cancer diagnosis or treatment. 

Age of onset for breast cancer patients with P/LP-Vs with P/LP-Vs variants and non-P/LP-Vs was not statistically significantly different, but for the respective mean age of onset, patients with the P/LP-Vs variant showed a younger in age of onset (33.7 years old) than the patients without the P/LP-Vs variant (34.8 years old). This is because all of the samples were young women. The triple negative characteristics were shown to be statistically significant differences in the non-P/LP-Vs variant group. In the P/LP-Vs group, a statistically significant difference was found in the higher percentage of Luminal B status than in the non-P/LP-Vs group. This result could be due to the fact that the number of patients with luminal B was twice as high as the triple negative patients.

## 4. Discussion

The probability of dying from breast cancer and distant metastases was considerably higher for BRCA1 and BRCA2 mutation carriers [26]. Many breast cancer patients who had a higher inherited risk of developing the disease tested negative for BRCA1 or BRCA2 P/LP-Vs, necessitating additional genetic testing using larger gene panels. Because of sequencing limitations, some individuals who test negative for BRCA1/2 P/LP-Vs may also have P/LP-Vs in other cancer susceptibility genes, such as TP53, PALB2, ATM, PTEN, STK11, and others, in 3–4% cases [3,27]. For this reason, the usage of multi-gene panels encompassing multiple susceptibility genes beyond BRCA1/2 is increasing because of the advancements made by next-generation sequencing (NGS) technology, which have transformed clinical approaches to genetic testing [6,28].

In this study, we identified 35 P/LP-Vs in the 13 established hereditary breast cancer genes (Table 2). Although ClinVar has registered many pathogenic variants in those 13 genes, only 20% (7/35) of P/LP-Vs have been registered. BRCA2 genes were found to be the most predominant genes with P/LP-Vs. In potentially pathogenic VUS variants, we also found 31 variants in 15 genes, and VUS variants in the MSH6 gene were found to be predominant among the others. Unfortunately, we did not have the recurrence data from all of the patients, so we could not analyze the impact of P/LP-Vs in BRCA1/2 genes with recurrence. However, in several previous studies, P/LP-Vs in BRCA1/2 genes could drive recurrence [5,29,30,31].

Previous studies held in the Asian region showed that the prevalence of P/LP-Vs of BRCA1/2 genes among women of a young age of onset and with familial history were 8.8% to 26.7% (Table 4). In our result, the variant rate of BRCA1/2 in familial breast cancer (FBC) samples was 44.4% (8/18). Overall, our variant rate of P/LP-Vs of BRCA1/2 in FBC was higher than previous studies findings (Table 4). Howver, the discrepancy regarding the high percentage in these studies may in part result from limitations due to small sample sizes. 

In our study, the P/LP-Vs group showed statistically significant difference with the non-P/LP-Vs group in Luminal B (*p* < 0.05), which might be because of P/LP-Vs that were found to be predominant in the *BRCA2* gene. This result was in line with previous studies that showed that approximately 75% of BCs in *BRCA2* PV carriers were more often Luminal B. On the other hand, TNBCs were found to make up around 70% of BCs developing in *BRCA1* PV carriers [36,37,38]. 

Patients with P/LP-Vs, such as in BRCA1/2, BRIP1, ATM, PALB2, and ATM genes, were predicted to benefit from the PARP inhibitor (Talazoparib or Olaparib). Cancer cells with harmful mutations in breast cancer susceptibility genes 1 or 2 (BRCA1/2) lack the ability to repair DNA double strand breaks, making these tumors heavily reliant on the single strand break repair pathway. The poly(adenosine diphosphate-ribose) polymerase (PARP) enzyme controls this pathway. Inhibition of PARP results in cell death in BRCA1/2 mutant cells as a result of the accumulation of irreparable DNA damage [39].

The CDKN2A gene is a gene that encodes two proteins, p16INK4A and p14ARF, which regulate cell growth and survival. One of our patients that had P/LP-Vs in the CDKN2A gene may have benefited from CDK4/6 inhibitors. Laboratory data suggest that cancer cells with loss-of-function alterations of CDKN2A may be sensitive to CDK4/6 inhibitors such as Palbociclib, Ribociclib, and Abemaciclib. In ER+ and HER2-amplified cell lines, Palbociclib was shown to be synergistic with Tamoxifen and Trastuzumab, respectively. In cell lines that had conditioned resistance to ER blocking, Palbociclib increased sensitivity to Tamoxifen [23].

One of the genes in cancer that is most frequently altered is PTEN, a lipid and protein phosphatase. Many malignancies, including breast cancer, frequently lose PTEN function. Preclinical research has shown that the essential lipid kinase that predominantly regulates PI3K pathway activation, cell proliferation, and survival in PTEN-deficient tumor cells is the PI3Kβ isoform (containing the p110β catalytic subunit). Related to variants in the PTEN gene, we found that two patients with P/LP-Vs could be sensitive to beta-isoform selective PI3K-targeted inhibitors, such as the investigational agents GSK2636771 and AZD8186 [25].

Besides P/LP-Vs in several genes, there was a missense variant in the TP53 genes (p. P72R) that was found in 52 patients (69.3%). Variants of apoptosis-related genes, such as TP53 codon 72, are associated with the susceptibility or prognosis of solid tumors. The TP53 codon 72 variant was found to be a strong predictor of the pathologic response to neoadjuvant chemotherapy in breast cancer [40,41]. Based on this finding, we suggest that clinicians consider using genomic sequencing to identify the presence of variants in BRCA1/2 and other genes related to breast cancer, such as TP53, ATM, PTEN, and CDKN2A. Genomic sequencing could lead to precision medicine. For example, if there are BRCA1/2 pathogenic variants, clinicians could consider using a PARP inhibitor; if there is a P72R mutation in the TP53 gene, clinicians could avoid using 5-Fluorouracil and Cisplatin because of resistance prediction. The limitations of our study were related to the small number of breast cancer patients. Compared with breast cancer populations, our sample size was small, only around 2.6%. In Indonesia, genomic sequencing research is an expensive research field. We tried to explore genomic aspects in breast cancer, although with limited resources. In addition to this limitation, we realized that we had a bias number in the patient characterization distribution, for example, in familial history distribution (n = 18 of 75) and TNBC subtype (n = 18 of 75). So, this might have resulted in a high variant rate of P/LP-Vs compared with previous studies.

## 5. Conclusions

The P/LP-Vs found both in BRCA1/2 genes and non-BRCA genes may increase the risk of breast cancer and alter drug responses. The screening of multigene variants is suggested, rather than BRCA testing only. Prior knowledge of the germline variant status is important for optimal breast cancer diagnosis and therapy.

## Figures and Tables

**Table 1:** Demographic characteristics of the breast cancer patients.

Characteristics	Patients (n = 75)
Age of onset (years)	34
Breast cancer family history; n (%)	18 (24%)
Molecular subtype; n (%)	
Triple negative	14 (18.7%)
HER2/ER positive	10 (13.3%)
Luminal B	31 (41.3%)
Luminal A	20 (26.7%)
Metastatic status; n (%)	16 (21.3%)
Stage of breast cancer; n (%)	
I	13 (17.3%)
II	27 (36%)
III	19 (25.3%)
IV	16 (21.4%)

**Table 2:** Pathogenic and likely pathogenic germline variants (P/LP-Vs).

Gene	HGVSg	Protein Change	Type of Variant	dbSNP/ClinVar ID	Clinical Significance	OncoKB-db Targeted-Drug Prediction	Number of Carriers
**BRCA1**	17:g.43091349delinsTTTAAAGTGCAGCTTTTC	p.I1395Kfs	Frameshift	-	Likely-Pathogenic *	-	1
	17:g.43093581_43093582delinsT	p.I650Kfs	Frameshift	-	Likely-Pathogenic *	Olaparib, Talazoparib	1
	17:g.43093821_43093822delinsT	p.P570Qfs	Frameshift	-	Pathogenic *	Olaparib, Talazoparib	1
**BRCA2**	13:g.32316515_32316516delinsT	p.C19Sfs	Frameshift	-	Pathogenic *	Olaparib, Talazoparib	1
	13:g.32332277delinsGCATACAT	p.G267Afs	Frameshift	-	Likely-Pathogenic *	Olaparib, Talazoparib	1
	13:g.32333103delinsTA	p.H543Tfs	Frameshift	-	Likely-Pathogenic *	Olaparib, Talazoparib	1
	13:g.32338277delinsGACTTTGACAGAAA	p.E1308Dfs	Frameshift	-	Likely-Pathogenic *	Olaparib, Talazoparib	1
	13:g.32340935_32340951delinsA	p.G2195Ffs	Frameshift	-	Pathogenic *	Olaparib, Talazoparib	1
	13:g.32340959delinsGATGA	p.V2203Efs	Frameshift	-	Likely-Pathogenic *	Olaparib, Talazoparib	1
	13:g.32379885delinsCA	p.T3033Nfs	Frameshift	rs397507419	Pathogenic	Olaparib, Talazoparib	21
	13:g.32398375_32398376delinsA	p.T3288Nfs	Frameshift	-	Pathogenic *	Olaparib, Talazoparib	1
**TP53**	17:g.7674917delinsTC	p.P72R	Nonsense	-	Pathogenic *	-	2
**STK11**	19:g.1219400_1219456delinsT	p.X153_splice	Splice-site	-	Pathogenic *	-	10
**PTEN**	10:g.87965294delinsTCTTATCA	p.Y346Lfs	Frameshift	-	Pathogenic *	GSK2636771, AZD8186	1
	10:g.87965293_87965297delinsC	p.L345Pfs	Frameshift	-	Pathogenic *	GSK2636771, AZD8186	1
**BRIP1**	17:g.61683605_61683606delinsA	p.N1147Mfs	Frameshift	-	Pathogenic *	Olaparib	2
**ATM**	11:g.108227881_108227882delinsG	p.F61Lfs	Frameshift	-	Pathogenic *	Olaparib	1
	11:g.108245025_108245026delinsA	p.G301fs	Frameshift	-	Pathogenic *	Olaparib	1
	11:g.108282707delinsCATACAACACTAAAAAATG	p.X1193_splice	Splice-site	-	Pathogenic *	Olaparib	1
	11:g.108302968_108302969delinsC	p.L1814Wfs	Frameshift	-	Pathogenic *	Olaparib	1
	11:g.108326058delinsCCTTCTTCCAACAGAAACGATTGT	p.L2270Pfs	Frameshift	-	Pathogenic *	Olaparib	1
	11:g.108329023delinsACTACAGGTTTTTTTGTTGTT	p.V2365Lfs	Frameshift	-	Pathogenic *	Olaparib	1
	11:g.108329022delinsCCCAGGGTGTCATTCACCCT	p.V2365Qfs	Frameshift	-	Pathogenic *	Olaparib	1
	11:g.108345760delinsTCAGTAGCTCAAGGG	p.F2813Qfs	Frameshift	-	Pathogenic *	Olaparib	1
**PALB2**	16:g.23635659_23635660delinsA	p.M296 *	Nonsense	143979	Pathogenic	Olaparib	4
	16:g.23629919_23629925delinsT	p.Y743 *	Nonsense	-	Likely-Pathogenic *	Olaparib	1
**MSH6**	2:g.47803500delinsAC	p.F1088Sfs	Frameshift	rs267608078	Pathogenic	-	4
	2:g.47803657_47803658delinsT	p.G1139Afs	Frameshift	rs587781544	Pathogenic	-	1
	2:g.47806453delinsCTTAGAT	p.C1269 *	Nonsense	-	Pathogenic *	-	2
**PMS2**	7:g.5987583_5987584delinsC	p.K394Sfs	Frameshift	rs1554298067	Pathogenic	-	1
	7:g.5987525delinsCT	p.D414Rfs	Frameshift	rs267608159	Pathogenic	-	5
	7:g.5987525_5987526delinsC	p.D414Tfs	Frameshift	-	Pathogenic *	-	6
**CDKN2A**	9:g.21974732_21974737delinsC	p.L31Gfs	Frameshift	-	Pathogenic *	Palbociciclib, Ribociclib, Abernaciclib	1
**RAD51C**	17:g.58734130delinsAATCCAGGAAATGCAGAAGAG	p.R347Nfs	Frameshift	-	Pathogenic *	Olaparib	1
**RAD50**	5:g.132595759_132595760delinsT	p.K722Rfs	Frameshift	rs397507178	Pathogenic	-	6

*: predicted by VarSome.

**Table 3:** Cancer characteristics of breast cancer patients and their correlation with pathogenic and likely pathogenic variants (P/LP-Vs) (*N* = 75).

		Non-P/LP-Vs Group *N* = 25	P/LP-Vs Group	
			*N* = 50	*p*-Value *
Mean of age of onset (SD)		34.8 (4.7)	33.7 (4.4)	0.4882
		*N* (%)	*N* (%)	
Family history	Yes	7 (28)	11 (22)	0.3272
	No	18 (72)	39 (78)	
Metastatic status	Yes	7 (28)	9 (18)	0.0929
	No	18 (72)	41 (82)	
Triple negative	Yes	7 (28)	7 (14)	**0.0151**
	No	18 (72)	43 (86)	
HER2 overexpression	Yes	4 (16)	6 (12)	0.4149
	No	21 (84)	44 (88)	
Luminal B	Yes	8 (32)	23 (46)	**0.0424**
	No	17 (68)	27 (54)	
Luminal A	Yes	6 (24)	14 (28)	0.5190
	No	19 (76)	36 (72)	

* *p*-values were calculated for P/LP-Vs group compared to Non-P/LP-VS group using Chi-square test for categorical variables and *t*-test for continuous variables (age of onset). The statistically significant difference values (<0.05) are shown in bold.

**Table 4:** Previous studies analyzing BRCA variants using NGS in young breast cancer patients.

Study	Number of Patients	Age of Onset	P/LP-Vs Variant Rate
Morocco			
Jouali et al. [32]	15	<50 years old	26.7%
Bakkach et al. [33]	82	<40 years old	16.7%
Palestine			
Hamameh et al. [34]	79	<40 years old	16.5%
Taiwan			
Wang et al. [5]	228	<40 years old	8.8%
Turkey			
Akcay et al. [35]	110	<45 years old	25.5%

## Data Availability

The raw data of all samples were deposited in the SRA under BioProject number: PRJNA606794 (https://www.ncbi.nlm.nih.gov/bioproject/?term=PRJNA606794 (accessed on 25 December 2021)).

## Supplementary Materials

The following supporting information can be downloaded at: https://www.mdpi.com/article/10.3390/diagnostics12092241/s1. Table S1: List of genes of TargetRichTM Hereditary Cancer Panel. Table S2: Distribution of P/LP-Vs in multiple P/LP-Vs carriers. Table S3: Variants of uncertain significance (VUS) found among the samples.
