# Supplementary material for: Targeted Sequencing of Germline Breast Cancer Susceptibility Genes for Discovering Pathogenic/Likely Pathogenic Variants in the Jakarta Population
Source: Diagnostics (Basel). 2022 Sep 16;12(9):2241. doi: 10.3390/diagnostics12092241 (PMC9498046; doi:10.3390/diagnostics12092241)
Supplement: Supplementary file 1 [file diagnostics-12-02241-s001.zip › diagnostics-1861531-supplementary.pdf]

**Table S1.** Gene List.

| Number | Gene |
|---|---|
| 1 | ATM |
| 2 | BRIP1 |
| 3 | MLH1 |
| 4 | NBN |
| 5 | RAD50 |
| 6 | TP53 |
| 7 | BARD1 |
| 8 | CDH1 |
| 9 | MRE11A |
| 10 | PALB2 |
| 11 | RAD51C |
| 12 | XRCC2 |
| 13 | BRCA1 |
| 14 | CHEK2 |
| 15 | MSH2 |
| 16 | PMS2 |
| 17 | RAD51D |
| 18 | BRCA2 |
| 19 | EPCAM |
| 20 | MSH6 |
| 21 | PTEN |
| 22 | RINT1 |

**Table S2.** Distribution of P/LP-Vs in multiple P/LP-Vs carriers.

| Carriers ID | Genes | HGVSg |
|---|---|---|
| brca14_rscm_17 | ATM | 11:g.108282707delinsCATACAACACTAAAAAATG |
| | BRCA2 | 13:g.32333103delinsTA |
| | MSH6 | 2:g.47806453delinsCTTAGAT |
| brca25_rscm_17 | BRCA2 | 13:g.32379885delinsCA |
| | STK11 | 19:g.1219400_1219456delinsT |
| brca28_rscm_17 | BRCA2 | 13:g.32379885delinsCA |
| | PMS2 | 7:g.5987525_5987526delinsC |
| brca37_rscm_17 | BRCA2 | 13:g.32332277delinsGCATACAT |
| | CDKN2 | 9:g.21974732_21974737delinsC |
| | RAD50 | 5:g.132595759_132595760delinsT |
| | TP53 | 17:g.7674917delinsTC |
| brca48_rscm_17 | PMS2 | 7:g.5987525_5987526delinsC |
| | PTEN | 10:g.87965294delinsTCTTATCA |
| **brca58_rscm_17** | PALB2 | 16:g.23635659_23635660delinsA |
| | PMS2 | 7:g.5987525delinsCT |
| brca59_rscm_17 | BRCA1 | 17:g.43093581_43093582delinsT |
| | MSH6 | 2:g.47803500delinsAC |
| | RAD50 | 5:g.132595759_132595760delinsT |
| | RAD51 | 17:g.58734130delinsAATCCAGGAAATGCAGAAGAG |
| brca67_rscm_17 | MSH6 | 2:g.47803500delinsAC |
| | PALB2 | 16:g.23635659_23635660delinsA |
| | RAD50 | 5:g.132595759_132595760delinsT |
| brca72_rscm_17 | BRCA1 | 17:g.43093821_43093822delinsT |
| | STK11 | 19:g.1219400_1219456delinsT |
| brca73_rscm_17 | ATM | 11:g.108245025_108245026delinsA |
| | BRIP1 | 17:g.61683605_61683606delinsA |
| | STK11 | 19:g.1219400_1219456delinsT |
| brca74_rscm_17 | BRCA2 | 13:g.32338277delinsGACTTTGACAGAAA |
| | PALB2 | 16:g.23635659_23635660delinsA |
| | TP53 | 17:g.7674917delinsTC |

| Carriers ID | Genes | HGVSg |
|---|---|---|
| **brca79_rscm_17** | BRCA1 | 17:g.43091349delinsTTTAAAGTGCAGCTTTTC |
| | PMS2 | 7:g.5987583_5987584delinsC |
| | STK11 | 19:g.1219400_1219456delinsT |
| **brca80_rscm_17** | ATM | 11:g.108345760delinsTCAGTAGCTCAAGGG |
| | BRCA2 | 13:g.32316515_32316516delinsT |
| | STK11 | 19:g.1219400_1219456delinsT |
| brca82_rscm_17 | BRCA2 | 13:g.32379885delinsCA |
| | PMS2 | 7:g.5987525_5987526delinsC |
| **brca85_rscm_17** | BRCA2 | 13:g.32379885delinsCA |
| | PMS2 | 7:g.5987525_5987526delinsC |
| | STK11 | 19:g.1219400_1219456delinsT |
| brca86_rscm_17 | ATM | 11:g.108326058delinsCCTTCTTCCAACAGAAACGATTGT |
| | BRCA2 | 13:g.32379885delinsCA |
| **brca89_rscm_17** | BRCA2 | 13:g.32379885delinsCA |
| | PMS2 | 7:g.5987525delinsCT |
| | STK11 | 19:g.1219400_1219456delinsT |
| brca90_rscm_17 | BRCA2 | 13:g.32379885delinsCA |
| | PMS2 | 7:g.5987525_5987526delinsC |
| brca91_rscm_17 | PALB2 | 16:g.23635659_23635660delinsA |
| | STK11 | 19:g.1219400_1219456delinsT |
| brca93_rscm_17 | PMS2 | 7:g.5987525delinsCT |
| | STK11 | 19:g.1219400_1219456delinsT |
| **brca98_rscm_17** | BRCA2 | 13:g.32379885delinsCA |
| | BRIP1 | 17:g.61683605_61683606delinsA |
| | MSH6 | 2:g.47803657_47803658delinsT |
| | RAD50 | 5:g.132595759_132595760delinsT |
| brca100_rscm_17 | BRCA2 | 13:g.32379885delinsCA |
| | PMS2 | 7:g.5987525_5987526delinsC |
| | RAD50 | 5:g.132595759_132595760delinsT |

**Carriers ID with bold text** have familial history (mother/aunt/grandmother) in breast cancer

**Table S3.** Potentially pathogenic VUS detected in breast cancer susceptibility genes.

| Gene | HGVSg | Type of variant | dbSNP / ClinVar ID | Number of carriers |
|---|---|---|---|---|
| BRCA1 | 17:g.43093594delinsCAAAA | Frameshift | - | 1 |
| BRCA2 | 13:g.32354874C>T | Missense | rs41293505 | 1 |
| STK11 | 19:g.1221967C>T | Missense | - | 1 |
| | 19:g.1226589G>A | Missense | rs775978755 | 1 |
| | 19:g.1222992C>T | Missense | rs750366043 | 1 |
| | 19:g.1219346G>A | Missense | rs567769257 | 1 |
| ATM | 11:g.108247122C>T | Missense | 950587 | 1 |
| | 11:g.108271274G>A | Missense | rs749471737 | 1 |
| | 11:g.108335101C>T | Missense | rs1591192429 | 1 |
| PALB2 | 16:g.23624080C>G | Missense | rs372931676 | 1 |
| BRIP1 | 17:g.61808594C>T | Missense | rs758360637 | 1 |
| | 17:g.61799232C>T | Missense | rs786202780 | 1 |
| | 17:g.61799193C>T | Missense | rs772570870 | 1 |
| | 17:g.61780973T>C | Missense | rs1567813893 | 1 |
| CHEK2 | 22:g.28725253C>T | Missense | rs587781667 | 1 |
| BARD1 | 2:g.214781051A>T | Missense | rs776157713 | 1 |
| | 2:g.214730494G>T | Missense | rs1553612535 | 1 |
| | 2:g.214728819G>A | Missense | rs76744638 | 3 |
| MLH1 | 3:g.36993576G>T | Missense | rs777971423 | 1 |
| | 3:g.37028831C>T | Missense | rs532873141 | 1 |
| MSH2 | 2:g.47414338C>G | Missense | 953109 | 3 |
| | 2:g.47414418G>T | Missense | rs587779197 | 2 |
| MSH6 | 2:g.47798716A>T | Missense | rs762168786 | 1 |
| | 2:g.47799065G>A | Missense | rs63750440 | 1 |
| | 2:g.47799639T>A | Missense | rs745937181 | 2 |
| | 2:g.47800636A>G | Missense | rs587782593 | 1 |
| | 2:g.47806650C>T | Missense | rs773763465 | 3 |
| PMS2 | 7:g.5999217C>T | Missense | rs876658387 | 1 |
| RAD51C | 17:g.58703259G>A | Missense | rs200857129 | 2 |
| RAD51 | 17:g.58703259G>A | Missense | rs200857129 | 1 |
| MUTYH | 1:g.45333414C>T | Missense | rs587782683 | 1 |
| | 1:g.45331466C>T | Missense | rs748700385 | 1 |
